# A large type IV-A choledochal cyst mimicking hydatid cyst of the liver: A case report

**DOI:** 10.1016/j.ijscr.2025.110898

**Published:** 2025-01-15

**Authors:** Fitsum A. Gemechu, Michael A. Negussie, Messay Gebrekidan, Biruk Zenebe Bekele, Elsa Wolde Mamo, Shimelis Nigussie Gebremariam

**Affiliations:** a School of Medicine, College of Health Sciences, Addis Ababa University, Addis Ababa, Ethiopia; b Department of Radiology, Menelik II Comprehensive Specialized Hospital, Addis Ababa, Ethiopia; c Department of Surgery, College of Health Sciences, Addis Ababa University, Addis Ababa, Ethiopia

**Keywords:** Choledochal cyst, Obstructive jaundice, MRCP, Case report

## Abstract

**Introduction:**

Choledochal cysts are rare congenital anomalies of the bile ducts, with adult presentations being uncommon. This case is notable for its atypical presentation in a young adult, mimicking a hydatid cyst in a region where echinococcosis is endemic.

**Case presentation:**

A 22-year-old female presented with a 3-month history of progressive jaundice, accompanied by 5 months of epigastric and right upper quadrant pain, dark urine, pale stools, pruritus, and significant weight loss. She reported a prior admission for cholangitis, treated with antibiotics. Examination revealed stable vital signs, icteric sclerae, right upper quadrant tenderness, and scratch marks on the skin. Laboratory investigations showed elevated liver enzymes and hyperbilirubinemia (total bilirubin = 26 mg/dL, direct bilirubin = 20.5 mg/dL). Initial imaging studies, including ultrasound and CT, suggested a hydatid cyst of the liver. However, MRCP revealed dilated intrahepatic and extrahepatic bile ducts, consistent with a Type IV-A choledochal cyst. The patient underwent cholecystectomy, extrahepatic bile duct excision, and Roux-en-Y cysto-jejunostomy. Histopathological analysis confirmed the diagnosis without evidence of malignancy. She recovered uneventfully, with no complications reported during a 6-month follow-up.

**Discussion:**

This case highlights the diagnostic challenges in differentiating choledochal cysts from hydatid cysts, particularly in endemic regions. The use of MRCP was pivotal in achieving an accurate diagnosis and guiding definitive management. Early surgical intervention minimized the risks of complications and malignancy.

**Conclusion:**

Type IV-A choledochal cysts can present atypically, mimicking hydatid cysts. Advanced imaging, especially MRCP, is critical for accurate diagnosis and management.

## Highlights

• Type IV-A choledochal cyst mimicked a hydatid cyst in a 22-year-old female with obstructive jaundice and systemic symptoms.
• MRCP confirmed the diagnosis, guiding surgical management with cholecystectomy and Roux-en-Y cysto-jejunostomy.
• Histopathology revealed no malignancy, confirming Type IV-A choledochal cyst.
• Postoperative recovery was uneventful, with no complications or recurrence at six months.

## 1 Introduction

Choledochal cysts are rare congenital malformations of the bile tract, most commonly involving the common hepatic duct, and are characterized by abnormal cystic enlargement of the biliary tree [1]. They are typically identified in childhood, with approximately 80 % of cases diagnosed early. Adult presentations are rare and are often associated with complications, including cholangitis, choledocholithiasis, cyst rupture, secondary biliary cirrhosis, obstructive jaundice, and an increased risk of bile duct cancer [2]. Todani (1977) classified choledochal cysts into five types, with Type IA being the most common, typically presenting in children, and Type IV-A being the second most common [1]. This case report discusses a 22-year-old female patient with a large Type IV-A choledochal cyst mimicking a hydatid cyst of the liver and its successful surgical management.

This case has been reported in line with the SCARE criteria [3].

## 2 Case presentation

A 22-year-old female presented to the surgical outpatient department (OPD) with a 3-month history of intermittent light yellowish discoloration of her eyes. Two weeks prior to her visit, the discoloration became deep and constant. She also reported a 5-month history of epigastric and right upper quadrant pain, which was colicky in nature, worsened with food intake, and radiated to her back. Additionally, she experienced intermittent dark urine, clay-colored stools, generalized skin itching, loss of appetite, and significant, unquantified weight loss. She denied fever or any relevant family history. The patient recalled one prior admission to the Emergency Department for cholangitis, which was treated with antibiotics.

On physical examination, her vital signs were stable, with icteric sclerae. Abdominal examination revealed tenderness on deep palpation of the right upper quadrant. On integumentary system examination, scratch marks were noted across her body. Other physical examination findings were unremarkable. Laboratory tests showed elevated liver enzymes (ALT = 88 U/L, AST = 60 U/L, ALP = 280 U/L), elevated total bilirubin (26 mg/dL), and direct bilirubin (20.5 mg/dL). Additional laboratory results are provided in Table 1.

**Table 1:** Patient's lab results with reference ranges.

Test	Results	Reference range	Units
Hemoglobin	13.5	12.0–16.0	g/dl
MCV	92	80–100	fL
WBC	8200	4000-11,000	cells/μL
Neutrophils (%)	68 %	40 % - 75 %	%
AST	60	0–32	U/L
ALT	88	0–35	U/L
ALP	280	35–104	U/L
Total Bilirubin	26	0.1–1.2	mg/dL
Direct Bilirubin	20.5	0.0–0.3	mg/dL
Na+	132	135–145	mmol/L
Cl-	94.5	96–106	mmol/L
K+	3.7	3.5–5.0	mmol/L
BUN	31.3	7–20	mg/dL
Cr	0.5	0.6–1.3	mg/dL
Amylase	73	30–110	U/Lit
Lipase	89	70–140	U/Lit
PT	13.7	11.4–17.7	Seconds
aPTT	27.1	24.2–36.3	Seconds
INR	1.16	1.0	Ratio
Albumin	3.7		
PITC	Non-reactive		
HBsAg	Negative		
HCV	Negative		

An abdominal ultrasound revealed multiple thin-walled cysts in the right lobe and hilum of the liver, the largest measuring 8 × 7 cm, suggesting a hydatid cyst of the liver. A subsequent CT scan showed a unilocular cystic mass in segment 4 of the liver, measuring 8 × 7.6 cm, with no internal septations. The mass compressed the common hepatic duct, with tortuous dilation and multiple cystic dilations of the intrahepatic biliary ducts, leading to a preliminary diagnosis of hydatid cyst of the liver with intraductal rupture.

**Fig. 1:**
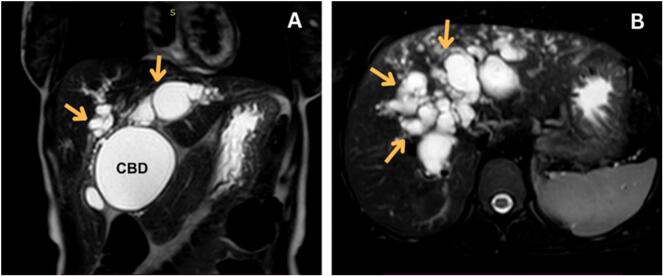
Coronal (A) and axial (B) T2-weighted MRI images demonstrating dilated intrahepatic and extrahepatic biliary ducts (arrows), with marked dilation of the common bile duct (CBD).

**Fig. 2:**
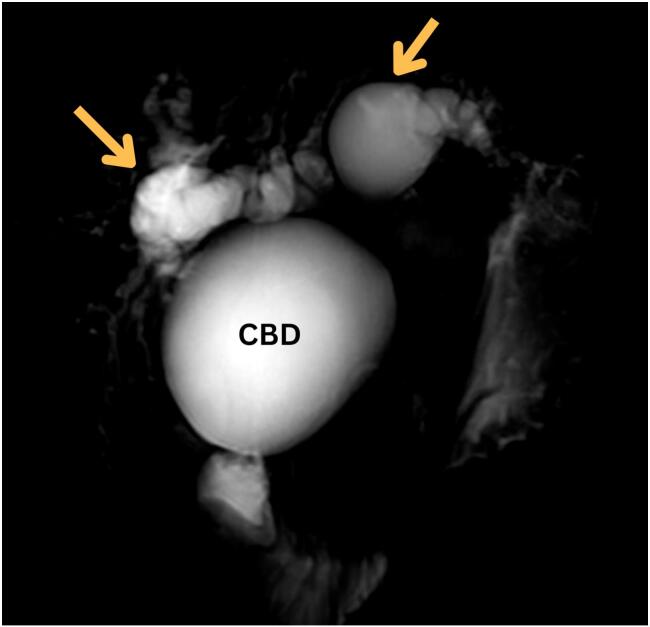
MRCP demonstrating dilated intrahepatic and extrahepatic biliary ducts (arrows), with marked dilation of the CBD and central dot signs, consistent with a Type IV-A choledochal cyst.

Due to diagnostic uncertainty, an MRCP was performed. MRCP revealed dilated intrahepatic and extrahepatic biliary ducts, including the common hepatic duct, cystic duct, and common bile duct (CBD), with the CBD being the largest. The dilation spared segment 7. Central dot signs were noted, indicating dilated intrahepatic bile ducts surrounding portal vein branches suggesting a type IV-A choledochal cyst. No filling defects or biliary stenosis were identified. The gallbladder was free of abnormalities, and the ampullary region and pancreatic ducts appeared normal (Fig. 1, Fig. 2).

With a preoperative diagnosis of a type IV-A choledochal cyst, the patient was taken to the OR with informed consent. The abdomen was entered through a right subcostal incision. Intraoperative findings revealed a hugely dilated extrahepatic bile duct extending from the junction of the right and left hepatic ducts to the intrapancreatic portion. The liver appeared bluish but without any palpable or visible masses. The dilated extrahepatic bile duct was carefully dissected from the pancreas and portal vein. At the liver hilum, the cyst involved both the right and left hepatic ducts. A cholecystectomy was performed, and the cyst was opened to identify the trifurcation of the hepatic ducts. The bile duct cyst was resected distal to this point. A Roux-en-Y cysto-jejunostomy was performed in an end-to-side fashion, followed by a side-to-side jejunojejunostomy. The peritoneal cavity was lavaged with warm saline, and a subhepatic drain was placed. The abdomen was closed in layers, and biopsy specimens were sent for histopathological analysis. The patient was transferred to the post-anesthesia care unit (PACU) with stable vital signs.

Histopathological analysis of the excised cyst revealed a biliary epithelial lining with areas of fibrosis and mild chronic inflammation, consistent with a type IV-A choledochal cyst. No evidence of dysplasia or malignancy was identified.

The patient remained in the ward for 13 days postoperatively, during which she received routine postoperative care and monitoring. She demonstrated steady improvement without any complications or complaints and was subsequently discharged in stable condition. Over the following 6 months, she was regularly followed up in the OPD, during which she reported no new complaints or complications, and her recovery remained uneventful.

## 3 Discussion

The term choledochal cyst refers to a range of congenital biliary tract abnormalities, previously known as idiopathic common hepatic duct dilatation, characterized by abnormal dilation of the biliary ducts [4,5]. Choledochal cysts are uncommon, occurring in 1:100,000 to 1:150,000 people, and are more prevalent in Asian populations (approximately 1:13,000 individuals). Although they are commonly diagnosed in the pediatric age group, choledochal cysts can present for the first time in adults in 20 % of cases [1,6]. While multiple theories have been proposed to explain their development, the most widely accepted is the abnormal union of the pancreaticobiliary duct [8].

Choledochal cysts are classified into five subtypes according to the Todani classification. Type I bile duct cysts consist of Type I-A (cystic dilation of the CBD), Type I—B (saccular dilation of the CBD), and Type I—C (fusiform dilation extending to the common hepatic duct). Type II cysts are extrahepatic duct diverticula. Type III cysts involve cystic dilation of the intraduodenal portion of the common bile duct (choledochocele). Type IV is subdivided into Type IV-A, characterized by common bile duct cysts with intrahepatic bile duct dilation, and Type IV-B, which involves multiple extrahepatic bile duct cysts without intrahepatic bile duct dilation. Type V is isolated intrahepatic cystic disease (Caroli disease) [9]. In our patient, MRCP findings revealed dilated intrahepatic and extrahepatic biliary ducts with a large cystic dilation in the common bile duct, consistent with a Type IV-A choledochal cyst.

The diagnosis and classification of choledochal cysts are established through imaging studies, including ultrasonography (USG), magnetic resonance imaging (MRI) with magnetic resonance cholangiopancreatography (MRCP), and computed tomography (CT). USG is often the preferred initial imaging method for assessing the intrahepatic and extrahepatic bile ducts, as well as the gallbladder, but it may not always clearly identify the cyst's exact origin within the bile duct [1]. MRCP is regarded as the diagnostic gold standard, with a sensitivity of 90–100 % for detecting biliary cysts [9,10].

In our case, the findings strongly suggest that the level of obstruction was at the CBD. The patient presented with classic symptoms of obstructive jaundice, including jaundice, dark urine, pale stools, and pruritus, alongside significantly elevated bilirubin levels, which are consistent with bile flow obstruction at the level of the CBD. Imaging studies, particularly MRCP, revealed dilated intrahepatic and extrahepatic bile ducts, with the CBD being the most prominently dilated. The presence of the central dot sign, indicating dilated intrahepatic bile ducts surrounding portal vein branches, further supported the likelihood of obstruction at the CBD. Intraoperative findings also aligned with this, showing a hugely dilated extrahepatic bile duct extending from the hepatic duct junction to the intrapancreatic portion of the CBD.

In regions where echinococcosis is endemic, cystic liver lesions are often presumed to be hydatid cysts, which can lead to misdiagnosis of other conditions with similar presentations, such as Type IV-A choledochal cysts. A study conducted at the University of Istanbul, Cerrahpasa Medical Faculty, reviewed 12 patients diagnosed with choledochal cysts between 1981 and 2000. The study found that three patients (25 %) were initially operated on under the assumption they had liver hydatid cysts; however, intraoperative findings revealed these were actually choledochal cysts [11]. In our patient, the initial ultrasound and CT findings were suggestive of a hydatid cyst of the liver. However, further imaging with MRCP established the correct diagnosis of a Type IV-A choledochal cyst, highlighting the diagnostic challenges in such cases.

Choledochal cysts can result in several complications, including cholecystitis, biliary strictures, cholangitis, recurrent pancreatitis, cholelithiasis, choledocholithiasis, and, occasionally, malignancy. It is particularly important to monitor adults with choledochal cysts, as they have a higher risk of biliary malignancy compared to children. Although there is a risk of cancer development at the residual stump following resection, removal of the cyst significantly reduces this risk [7].

The recommended management for choledochal cysts is definitive surgical intervention. In a study by Richa et al. involving 35 patients with Type IV-A choledochal cysts, removal of the extrahepatic cyst with drainage of the intrahepatic portion through a wide hilar or sub-hilar anastomosis showed excellent outcomes. For cases with symptomatic intrahepatic involvement, such as cholangitis or biliary cirrhosis, treatment protocols similar to those for Type V cysts are followed, which may involve hepatic resection for localized disease or liver transplantation for widespread involvement [10]. While hepaticoduodenostomy and Roux-en-Y hepaticojejunostomy are both viable options for biliary reconstruction, the latter is generally preferred [12]. In our patient, a cholecystectomy with extrahepatic bile duct excision and Roux-en-Y cysto-jejunostomy was performed, resulting in excellent postoperative outcomes, as evidenced by the patient's uneventful recovery and lack of complications during the 6-month follow-up period.

## 4 Conclusion

Diagnostic challenges are often faced in differentiating choledochal cysts from other cystic liver lesions, such as hydatid cysts. This is especially true in regions where echinococcal infection is prevalent. This case marks the importance of advanced imaging techniques, particularly MRCP, in accurately identifying and classifying choledochal cysts to guide appropriate management.

## CRediT authorship contribution statement

**Fitsum A. Gemechu**: Writing – original draft, Conceptualization, Resources. **Michael A. Negussie**: Writing – original draft, Conceptualization, Resources. **Messay Gebrekidan**: Writing – review & editing, Data curation. **Biruk Zenebe Bekele**: Writing – review & editing, Resources. **Elsa Wolde Mamo**: Writing – review & editing. **Shimelis Nigussie Gebremariam**: Supervision.

## Ethical approval

Ethical approval for this case report was provided by the Department of Surgery, College of Health Sciences, Addis Ababa University, Addis Ababa, Ethiopia (ethical approval number was not provided).

## Guarantor

Dr. Michael A. Negussie

## Consent for publication

Written informed consent was obtained from the patient for publication of this case report and accompanying images. A copy of the written consent is available for review by the Editor-in-Chief of this journal on request.

## Funding

No source of funding is provided for this case report.

## Registration of research studies

N/A.

## Declaration of competing interest

The authors declare that they have no known competing financial interests or personal relationships that could have appeared to influence the work reported in this paper.

## References

[1] Khandelwal C., Anand U., Kumar B., Priyadarshi R.N. (2012). Diagnosis and management of choledochal cysts. Indian J. Surg..

[2] de Albuquerque V., De Macedo F.P., Costa K.G. (2020). Choledochal cyst—unusual presentation in the adult phase: case report. Int. J. Surg. Case Rep..

[3] Sohrabi C., Mathew G., Maria N., Kerwan A., Franchi T., Agha R.A. (2023). The SCARE 2023 guideline: updating consensus surgical CAse REport (SCARE) guidelines. Int J Surg Lond Engl..

[4] Bandi Babita G, Prathamesh Vijay Kotawadekar, Pradeep S. Patil. Type IV-A choledochal cyst in newborn. Int J Sci Res. 2023;12(4):1–2. doi:10.36106/ijsr/1234567.

[5] Dewi D.K., Kurniawan O., Gunawan D.I., Nugraha H.G. (2025). A case of choledochal cyst type IV. Radiol Case Rep..

[6] Liu C.L., Fan S.T., Lo C.M., Lam C.M., Poon R.T., Wong J. (2002). Choledochal cysts in adults. Arch. Surg..

[7] Kim J.Y., Kim H.J., Han H.Y. (2015). A case report of an unusual type of choledochal cyst with choledocholithiasis: saccular dilatation of the confluent portion of both intrahepatic ducts. J Korean Soc Radiol..

[8] Çağan Appak Y., Günşar C., Doğan G., Tarhan S., Kasırga E. (2017). The association of choledochal cyst and pancreatitis: a case report and review of the literature. J Pediatr Res..

[9] Ali A., Gomes R.R. (2021). A case of choledochal cyst complicated by acute pancreatitis with choledocholithiasis. J Gastroenterol Hepatol Res..

[10] Metcalfe M.S., Wemyss-Holden S.A., Maddern G.J. (2003). Management dilemmas with choledochal cysts. Arch. Surg..

[11] Durgun A., Gorgun E., Kapan M. (2002). Choledochal cysts in adults and the importance of differential diagnosis. J. Hepatobiliary Pancreat. Surg..

[12] Lal R., Agarwal S., Shivhare R. (2005). Type IV-A choledochal cysts: a challenge. J. Hepatobiliary Pancreat. Surg..

